# Registration of ovarian cancer in England and Wales

**DOI:** 10.1054/bjoc.2000.1256

**Published:** 2000-06-15

**Authors:** Nicola MacDonald, Usha Menon, Ian Jacobs

**Affiliations:** St Bartholomew’s and Royal London School of Medicine and Dentistry, Queen Mary and Westfield College; Gynaecological Oncology, St Bartholomew’s Hospital, London, EC1A 7BE


**REFERENCES**

Macdonald N, Sibley K, Rosenthal A, Menon U, Jeyarajah A, Oram D and Jacobs I (1999) A comparison of national cancer registry and direct follow-up in the ascertainment of ovarian cancer. *Br J Cancer* **80**: 1826–1827

Office for National Statistics (1999) *Cancer statistics – registrations, England & Wales*, 1993. ONS Series MB1 No. 26. London: The Stationery Office

Parkin DM, Chen VW, Ferlay J, Galceran J, Storm H and Whelan S (1994) *Comparability and quality control in cancer registration.* IARC Technical Report No. 19. Lyons: IARC

Thames Cancer Registry (1997) *Report for 1996*. London: TCR

doi: 10.1054/ bjoc.2000.1256, available online at http://www.idealibrary.com on IDEAL®

**Sir**,

We read the letter from Quinn et al with interest and are glad to have an opportunity to acknowledge the cooperation received from the staff at the ONS throughout our study. The ONS team made considerable efforts to provide comprehensive reporting of ovarian cancer cases. Our report was not intended to be a criticism of the important work performed by the NHSCR.

We reported our comparison of ‘direct’ and NHSCR follow up for ovarian cancer in order to provide information for the design of future research studies. Although the two methods of follow up are complementary, direct follow up identified more cases of ovarian cancer and identified them in a shorter period of time than was possible via the NHSCR. Researchers need to be aware of the issues of incomplete registration and the delay in notification through the NHSCR and consider the option of using an additional method of follow up. These issues have major implications for the design of clinical trials and in this context we hope that the data provided by our study is of some value.

Quinn et al highlighted the limitations of ‘follow-up’ compared to ‘flagging’ studies via the NHSCR. Whilst these points are entirely valid they do not explain the eleven cases of ovarian cancer not identified by the NHSCR in our study. First, follow up was carried out by the NHSCR in 1997–98, a time point more than five years after diagnosis of the ovarian cancer cases in our study. Second, repeated searches were performed by the NHSCR for the eleven cases both manually and by computer. Although the data originally supplied to the NHSCR was incomplete for some study participants, complete data for the relevant eleven cases was resubmitted for additional searches once the discrepancy was identified. It is possible that flagging would eventually identify these cases but a delay of more than five years from study completion to analysis has major implications for a clinical trial. As noted by Quinn et al a total of four cases of ovarian cancer reported by the NHSCR were not identified by direct follow-up. However, the study was limited to cases diagnosed between 1986 and 1993 because this allowed a 5 year period for data collection by the NHSCR and was the period of direct follow. Two cases of ovarian cancer identified by the NHSCR but diagnosed after 1993 were not therefore reported in our paper. The same applies to three other cases of ovarian cancer diagnosed after 1993 but not identified by the NHSCR.

It seems sensible for researchers currently designing clinical studies requiring long-term follow-up to consider using direct follow up as well as flagging with the NHSCR. Direct follow up is a rapid and reliable means of identifying cancer cases which complements information provided by the NHSCR. Major efforts and numerous changes are being made in the cancer registration system which are improving the research value of this key resource. We strongly support investment in cancer registration and appreciate the efforts being made by staff in the regional cancer registries and at the ONS. Hopefully in the future the use of direct follow up in clinical trials will not be necessary!

*Nicola MacDonald, Usha Menon, Ian Jacobs*
*St Bartholomew’s and Royal London School of Medicine and Dentistry, Queen Mary and Westfield College*

*Ian Jacobs MD MRCOG Professor of Gynaecological Oncology*
*St Bartholomew’s Hospital, London EC1A 7BE*

© 2000 Cancer Research Campaign

